# Adult T-Cell Leukemia/Lymphoma with CLL-Like Morphology—A Case Report

**DOI:** 10.1155/2010/729790

**Published:** 2010-03-24

**Authors:** Nishant Tageja, Mobayed Mohammad, Gail Bentley, Carter Bishop

**Affiliations:** ^1^Department of Internal Medicine, Wayne State University/Detroit Medical Center (WSU/DMC), Detroit, MI 48201, USA; ^2^Division of Hematology/Oncology, Wayne State University/Karmanos Cancer Institute, Detroit, MI 48201, USA; ^3^Department of Pathology, Wayne State University/Detroit Medical Center (WSU/DMC), Detroit, MI 48201, USA

## Abstract

Adult T-cell Leukemia/Lymphoma (ATL) is rarely seen in the U.S. and Europe, usually limited to African Americans from the southeastern U.S. and immigrants from HTLV-1 endemic areas. Reaching an accurate and timely diagnosis of ATL in such nonendemic areas can be challenging, owing to limited exposure, diverse manifestations, and varying cell morphology. We present a case of chronic adult T-cell leukemia (ATL) with Chronic Lymphocytic Leukemia- (CLL-) like morphology that remained untreated for ten years and then developed treatment refractory acute ATL crisis.

## 1. Introduction

Adult T-cell leukemia/lymphoma (ATL) is a highly aggressive neoplasm of postthymic pleomorphic activated T lymphocytes, with a mean survival of 6–11 months [1]. Although endemic in southern Japan, the Caribbean, and Central/South America, HTLV-1 infection and ATL have only rarely been reported in the U.S. and Europe, usually limited to African Americans from the southeastern U.S. and immigrants from HTLV-1 endemic areas. The diversity of cell morphology in ATL is well known but its implication on overall clinical outcome has not been fully explored [2]. We present a case of chronic adult T-cell leukemia-lymphoma (ATL) with Chronic Lymphocytic Leukemia- (CLL-) like morphology that remained untreated for ten years and then developed treatment refractory Acute ATL crisis.

## 2. Case Presentation

A 57-year-old Caucasian man diagnosed with T-cell leukemia 10 years previously, presented in the outpatient clinic with worsening abdominal distension. A comprehensive review of systems revealed decreased appetite with a 30 lb weight loss over 4-5 weeks, nausea, vomiting, dry cough, and night sweats. He denied any history of intravenous drug abuse, blood transfusion, or travel outside the United States. On initial physical examination the patient was noted to have diffuse lymphadenopathy in the axillary and inguinal regions. Breath sounds were decreased diffusely and mild bibasilar crackles were noted. Cardiovascular exam was normal. Abdominal examination revealed moderate ascites with hepatosplenomegaly. He had a scaly macular skin lesion over his sacrum, measuring approximately 9 cm by 4 cm. 

The patient had been diagnosed at an outside facility with T-Pro Lymphocytic Leukemia (T-PLL) in 1998 based on leukocytosis with lymphocytosis, 21300, cu.mm and presence of relatively uniform lymphocytes with mild abnormality in the nuclear shape, prominent central nucleoli, and scant cytoplasm, expressing CD2 (97% cells), CD3 (93%), CD4 (92%), and CD5 (96%) on flow cytometry. CLL-like small mature lymphocyte morphology was observed in more than 60% of the cell examined. Sadly, ATL was not considered in the differential at the time of initial diagnosis. The patient stayed asymptomatic from his disease for over 10 years with no evidence of anemia, thrombocytopenia, splenomegaly, or palpable lymphadenopathy.

Initial laboratory results in our institution revealed leukocytosis 281,900 cu.mm with deranged LFTs, elevated LDH 330 mg/dl, albumin 1 gm/dl, BUN 31 mg/dl and calcium, 13.9 mg/dl. CT-abdomen revealed hepatosplenomegaly with generalized lymphadenopathy. Serology for Hepatitis A, B, and C was negative, as was the serology for human immunodeficiency viruses 1 and 2 (HIV1 and 2). Computed Tomography (CT) scans of chest, abdomen, and pelvis at our institution revealed generalized lymphadenopathy in the chest, abdomen and pelvis with enlarged liver and moderate splenomegaly. Skeletal survey revealed no lytic bone lesions. 

**Figure 1:**
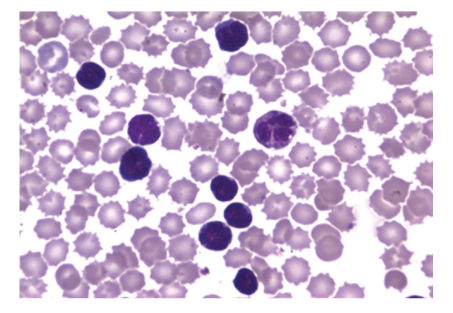
Peripheral blood smear showing leukemic cells with varying cell morphologies (including CLL-like small lymphocytes).

**Figure 2:**
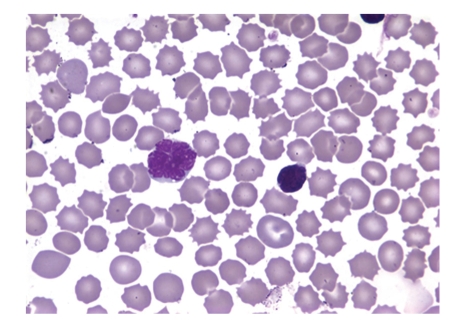
A Flower cell in the peripheral smear characteristic of ATL.

Peripheral blood smear showed medium to large lymphoid cells showing convoluted nuclei, coarsely clumped chromatin, and basophilic scant cytoplasm with floret forms, blasT-cells, and CLL-like cells (Figures 1 and 2). Flower cells with polylobulated nuclei were also found in the ascites fluid. Immunophenotyping revealed an abnormal T-cell population expressing CD2 (96% cells), CD4 (94%), CD5 (95%), and CD25 (81%) with absent TdT. CD7, a T-cell tumor marker which aids in differentiating ATLL from other T-cell malignancies, was also negative. EIA detected virus specific serum antibodies to HTLV-1 which were later confirmed by Western blotting, thus confirming the diagnosis of ATL. Trans-Thoracic Echocardiography revealed an ejection fraction of 35% with no regional wall motion abnormalities. The patient was started on chemotherapy with CEOP regimen (cyclophosphamide, etoposide, vincristine,and prednisone) but continued to deteriorate clinically, eventually succumbing to his disease within 3 months of treatment.

## 3. Discussion

The diagnosis of ATL in nonendemic regions is challenging and may be particularly difficult to establish. Clinical suspicion is usually based on a constellation of clinical features and laboratory investigations including lymphocyte morphology, immunophenotype, histology of the affected tissues and serology, or DNA analysis for HTLV-I. Organomegaly, skin involvement, circulating atypical lymphocytes (“flower” cells) with a CD4+ CD25+ phenotype, and hypercalcemia are the most common disease features at the time of presentation [3]. The differential diagnosis often includes other mature T-cellneoplasms such as T-cell prolymphocytic leukaemia (T-PLL), Sézary syndrome (SS), and peripheral T-cell lymphomas. 

The diverse clinical features of ATL have led to its subclassification into acute, lymphoma, chronic, and smoldering types [4]. The clinical courses of the chronic and smoldering types are indolent, conventional chemotherapy does not improve prognosis except in patients with an unfavorable chronic type who present with lactate dehydrogenase or blood urea nitrogen levels above the normal upper limits, and an albumin level below the normal lower limit [5]. Patients with acute transformation generally have a poor prognosis because of multidrug resistance of malignanT-cells and a large tumor burden with hypercalcemia and multiorgan failure. Approximately three-fourths of chronic-ATL patients die within 4 years of diagnosis [5]. Interestingly, our patient had an asymptomatic course for at least 10 years before presenting with acute crisis, which we believe is because of the unusual tumor morphology.

ATL cells in peripheral blood (PB) are usually characterized by having highly indented or lobulated nuclei with homogeneous and condensed chromatin, small or absent nucleoli, and agranular and basophilic cytoplasm. However, the diversity of morphology in ATL is well-established and several cases with peculiar morphological features have been reported. In addition to polylobed cells, some blastic cells with basophilic cytoplasm are almost always seen in blood films of ATL [6]. A few cases with presence of bizarre gianT-cells or large granular lymphocytes [7, 8] have also been reported, and shown to have aggressive clinical features and a poor prognosis. 

ATL with CLL-like morphology is rare but has been suggested to have a longer transformation-free survival in a Japanese study by Tsukasaki et al. [2]. It was reported that cases with >50% CLL-like cells (*n* = 7; all chronic type) were younger (53.1 ± 12.2 versus 66.9 ± 10.6 years; *P* = .038) and showed longer acute-crisis free survival (mean: 16.7 versus 3. 0 years; *P* = .012) than chronic cases with <50% CLL-like cells. The authors went on to suggest that CLL-like ATL maybe an earlier phase of ATL leukemogenesis would appear to be a good prognostic factor for chronic type. 

To conclude, the behavior of ATL in our case further substantiates the thought thaT-cell morphology is closely associated with genotype, phenotype, and clinical picture of ATL. The case also highlights the potential difficulties in reaching an accurate and timely diagnosis of ATL in nonendemic areas, owing to diverse manifestations and varying cell morphology. A close coordination between the pathologist and the clinician is vital in patients with rare disease pathology and unusual cell morphologies, as a misdiagnosis can have important clinical implications.

## Competing Interests

The author(s) declare that they have no competing interests.

## Authors' Contributions

All the authors contributed equally in drafting the manuscript and revising it critically for important intellectual content.
